# Hospital and Health Law in the Empire and U.S.A.

**Published:** 1915-05-22

**Authors:** 


### Hospital and Health Law in the Empire and U.S.A.

The science of comparative legislation has this use, among others, that it puts fresh suggestions into the mind, and shows how the varied conditions of different countries modify laws. Viewed in that light the latest volume issued by the Society of Comparative Legislation is full of interest, partial proof of which fact we now offer by briefly noting the laws made in other parts of the Empire and elsewhere during 1914 regarding hospitals and hospital administration.

### The Combined System in Cape Colony.

In Cape Colony, where an ordinance has been passed codifying existing hospital laws, the following financial provision has been made for the upkeep of public hospitals and kindred charitable institutions. Hospital boards are constituted for each district of the province, and these boards may be subsidised by the Government to the extent of 30s. for every £1 of the value of all voluntary contributions or gifts of money; £1 for every £1 of the value of all devises or bequests up to a maximum of £500; and £1 for every £1 received as fees from patients.

### Private Hospitals in Canada.

In Canada two Legislatures have made enactments which will interest doctors. In New Brunswick the Council of Physicians and Surgeons of that province are required to admit to registration, on payment of the registration fee, any person desirous of practising in New Brunswick who is registered by the General Medical Council of Great Britain. And in British Columbia the Hospitals Act of that province has been amended to the effect that henceforth private hospitals—*i.e.*, those receiving no provincial aid—are made subject to licensing and inspection, and obliged to keep a register of all patients treated.

### Labour Hospitals in Straits Settlements.

One of the laws passed by the Legislature of the Straits Settlements makes us think that even for this country the "Labour Hospital" may not be merely a dream. There it was felt that the hospital accommodation necessary for adequately attending to the needs of sick labourers on agricultural estates could be more economically, as well as more efficiently, provided if they were placed under one control in certain districts. At the same time it was not thought desirable to relieve estate owners of the liability cast on them by previous legislation of providing for the health of their labourers. Therefore it has been ordained that, in such parts of the Colony as may be suitable, the owners should be relieved of the duty of constructing and maintaining separate hospitals, but should contribute instead a rateable lump sum per cultivated acre for the purpose of constructing and equipping hospitals common to defined areas, and an annual rate towards their upkeep. The same summary legal measures as in the case of municipal rates are enacted for the enforcement of payment of this hospital rate.

### The Sterilisation of Criminals in U.S.A

Turning now to the United States, we find that North Dakota and Oregon have followed the example of several other States by enacting laws permitting the sterilisation of criminals and degenerates. In North Dakota, whenever the head of any State prison, reform school, State school for the feeble-minded, or asylum for the insane, shall certify, in writing, that he believes that the mental or physical condition of any inmate would be improved, or that procreation by such inmate would be likely to result in defective or feeble-minded children with criminal tendencies, it shall be lawful to perform a surgical operation for the sterilisation of such inmate in accordance with the Act. The Act then goes on to provide for a board of examiners consisting of the chief medical officer of the institution, the Secretary of the State Board of Health, and one competent physician and surgeon of at least ten years' standing. If this board find that the operation is advisable, they shall designate some surgeon to perform it. The Act further directs the chief medical officer, having charge of inmates who have been operated upon, to make careful observation of them with a view to ascertaining the effect of the operation upon the mental, moral, and physical condition of such sterilised person, and report thereon, at least once a year, to the Governor of the State. Apparently quick and important results are expected, for the Act has this emergency clause: "Whereas heredity plays a most important part in the transmission of crime, insanity, idiocy, and imbecility, and our institutions for degenerates are overcrowded . . . this Act shall take effect and be in force from and after its passage"!

